# Improving Resident Doctors Confidence in Navigating Pay, Rota and ARCP Requirements When Transitioning to Less Than Full Time Training at South West London and St George's NHS Trust

**DOI:** 10.1192/bjo.2026.11369

**Published:** 2026-06-30

**Authors:** Darshita Depala, Soracha Healy, Vinita Goveas

**Affiliations:** South West London and St George’s NHS Trust, London, United Kingdom

## Abstract

**Aims:**

To increase the confidence of resident doctors in navigating pay, rota and ARCPrequirements, related to LTFT training following a targeted educational intervention at the August 2025 induction.

**Methods:**

Around 25% of resident doctors in the UK now work LTFT (Less Than Full Time). However, many face issues with navigating training requirements, pay and rota arrangements.

A Quality Improvement (QI) approach was used to identify and address knowledge gaps among LTFT trainees.

A baseline questionnaire was first distributed to resident doctors at South West London and St George’s NHS Trust between June and July 2025 to identify common challenges. In response to these findings, a comprehensive induction presentation was delivered in August 2025 covering essential topics such as pay (nodal points, hourly rates, and flexible training premia), rotas (pro-rata leave and on-call obligations), and ARCP requirements (pro-rata assessments and adjusted completion dates). To evaluate the intervention’s impact, a post-induction questionnaire was subsequently administered to the August 2025 cohort of resident doctors at the Trust to measure improvements in trainee confidence levels.

**Results:**

Prior to the introduction of the Less Than Full Time (LTFT) induction session (June–July 2025), resident doctors reported low baseline confidence across all three areas of LTFT training evaluated, with mean scores of 2.3/5, 2.3/5 and 2.2/5 for pay, ARCP requirements and rota domains respectively.

Following the implementation of the LTFT induction session (August 2025), confidence levels showed a marked improvement among respondents. Mean confidence in navigating pay issues, ARCP requirement and the rota rose to 4.29/5, 3.9/5, and 4.3/5, representing an 86%, 68% and 95% increase from baseline respectively.

Qualitative feedback from the post-induction cohort was overwhelmingly positive, with 100% of trainees describing the presentation as “very clear and informative”.

**Conclusion:**

Providing a dedicated LTFT session during induction significantly improves resident doctors' confidence in navigating the administrative hurdles of non-standard training. We are developing further guidance for resident doctors to be published on the Trust’s intranet.

